# Gold–Graphene Quantum Dot Hybrid Nanoparticle for Smart Diagnostics of Prostate Cancer

**DOI:** 10.3390/bios14110534

**Published:** 2024-11-04

**Authors:** Divakar Raj, Arun Kumar, Dhruv Kumar, Krishna Kant, Ashish Mathur

**Affiliations:** 1 Allied Health Sciences, School of Health Sciences and Technology, University of Petroleum and Energy Studies, Dehradun 248007, Uttarakhand, India; divakar.107502@stu.upes.ac.in (D.R.); dhruv.kumar@ddn.upes.ac.in (D.K.); 2 Mahavir Cancer Sansthan, and Research Centre, Patna 801505, Bihar, India; drarunk31@gmail.com; 3 Department of Biotechnology, School of Engineering and Applied Sciences, Bennett University, Greater Noida 201310, Uttar Pradesh, India; 4 CINBIO, Universidade de Vigo, Campus Universitario Lagoas Marcosende, 36310 Vigo, Spain; 5 Centre for Interdisciplinary Research and Innovation (CIDRI), University of Petroleum and Energy Studies, Dehradun 248007, Uttarakhand, India

**Keywords:** prostate cancer, Au–GQD, electrodeposition, electrochemical biosensor, point-of-care detection

## Abstract

Prostate cancer is one of the most prevalent cancers afflicting men worldwide, often detected at advanced stages, leading to increased mortality rates. Addressing this challenge, we present an innovative approach employing electrochemical biosensing for early-stage prostate cancer detection. This study used Indium–Tin Oxide (ITO) as a substrate and a deposited gold–graphene quantum dot (Au–GQD) nanohybrid to establish electrochemical sensing platforms for DNA-hybridization assays. A capturing DNA probe, PCA3, was covalently immobilized on the surface of the Au–GQDs and deposited electrochemically onto the ITO electrode surface. The Au–GQDs enabled the capturing of the target PCA3 biomarker probe. The sensor achieved a limit of detection (LoD) of up to 211 fM and presented a linear detection range spanning 1 µM to 100 fM. A rapid 5-min response time was also achieved. The tested shelf life of the pre-immobilized sensor was approximately 19 ± 1 days, with pronounced selectivity for its intended target amidst various interferants. The sensing device has the potential to revolutionize prostate cancer management by facilitating early-stage detection and screening with enhanced treatment efficacy.

## 1. Introduction

Prostate cancer is a critical health concern worldwide. Typically generated as an outcome of interruption in expected cell signaling consequences [1], it develops slowly over a long period and is usually detected in late-stage diagnostics. Prostate cancer is the second most frequent cancer among males globally, after lung cancer. In 2020, there were an estimated 1.4 million new instances of prostate cancer worldwide, accounting for 7.3% of all new cancer cases and 3.75 lacs of deaths, which is projected to be around 2.43 million new cases and around 7.40 lacs of deaths by 2040 [2]. According to the World Health Organization (WHO), the number of new cancer patients is anticipated to increase by 70% in the upcoming two decades. Initially, the prostate cancer risk spikes with age, and it is more prevalent in males over the age of 50–60 years. Due to delayed diagnosis, the tumors typically develop into malignant tumors, which might have lethal consequences; early detection can increase the number of possible treatment options as well as increasing the likelihood of a good patient outcome and lowering the risk of complications that may result from more aggressive therapies [3,4]. Nowadays, most prostate malignancy surveillance is performed by calculating the prostate serum antigen (PSA) levels in blood samples. Serum PSA tests are pretty sensitive, but disappointingly, they are not precise for prostate cancer, as high PSA concentrations may not be cancer-related. Sometimes, prostatitis may lead to a high PSA level; therefore, the quantification of PSA and Digital Rectal Examination (DRE) may lead to unnecessary biopsies or overdiagnosis. However, PCA3 (Prostate Cancer Antigen 3) is a long non-coding RNA (lncRNA) biomarker with 3922 nucleotides and is mainly overexpressed only in prostate tumors without apparent connection to other prostate gland abnormalities [5]. It has been proven to be a dedicated biomarker for prostate cancer, demonstrated higher specificity than the PSA for prostate cancer diagnosis, and been found in non-invasive samples like blood and urine [6,7]. Reverse transcription polymerase chain reaction (RT-PCR) [5], fluorescence emissions, and transcription-mediated amplification (TMA) have often been used to identify PCA3 [8,9].

Due to its easy availability in non-invasive samples (urine), PCA3 has become an attractive biomarker for the non-invasive early detection of prostate cancer. The present diagnostic test includes the detection of PCA3 in post-DRE urine samples that were commercially developed and authorized for clinical use in the USA [10,11]. Quantitative nucleic acid amplification with excellent sensitivity and specificity is present in the Progensa test, which determines the presence and concentration of PCA3 biomarkers. Still, these tests are expensive, are time-consuming, and require skilled individuals for their operation [12]. Developing a rapid and sensitive approach toward point-of-care (PoC) development is crucial to overcome the present challenges. Incorporating nano-carbon materials significantly improves recognition sensitivity, leading to higher reliability and long-term usability for PoC. Because of their distinctive electrical, mechanical, and chemical characteristics, carbon nanomaterials such as carbon nanotubes (CNTs) and graphene have shown significant promise for application in electrochemical biosensors [13,14]. The use of Au–GQDs in electrochemical biosensors has various benefits whenever gold nanoparticles have excellent electrical conductivity, while the graphene quantum dots have large surface areas and are biocompatible [15,16,17,18]. In the present study, we have explored the application of PCA3 biomarkers for on-chip electrochemical sensor development, reducing the detection time and enhancing the sensitivity for prostate cancer detection in a cost-effective setting. Electrochemical biosensors offer outstanding sensitivity and assist in detecting analytes at the lowest concentrations. They can detect a wide range of analytes particular to the target [19].

**Figure 1:**
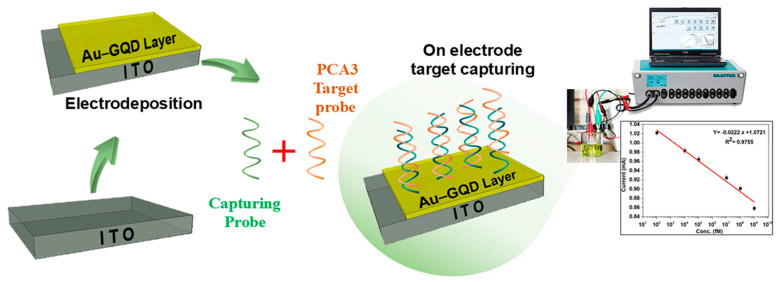
Schematic illustration of step-by-step fabrication of proposed sensor for PCA3 detection strategy.

Additionally, electrochemical biosensors are feasible for cost-effective testing in medical clinics due to their low cost, simplicity, ability to be miniaturized, quick response time, and stability. Electrochemical transduction technology has been used extensively in biosensing [20,21,22]. Herein, we used ITO glass electrodeposited with Au–GQDs, followed by surface immobilization ssDNA probes for capturing the target PCA3, characterized by electrochemical studies (cyclic voltammetry (CV) and electrochemical impedance spectroscopy (EIS)). The step-by-step fabrication of the proposed sensor is presented in Figure 1. The developed approach and methodology offer an exceptionally highly sensitive LoD, which is suitable for their application for the rapid and early-stage detection of prostate cancer.

## 2. Materials and Methods

ITO was purchased from Techinstro Pvt. Ltd., New Delhi, India (dimensions: L, 100 mm × W, 100 mm; thickness: 1.1 mm; resistivity: 20 ohms/sq.). D-Glucose (dextrose) was purchased from MERCK (Mumbai); tri-sodium citrate, sodium di-hydrogen orthophosphate (NaH_2_PO_4_), sodium di-sodium hydrogen orthophosphate (Na_2_HPO_4_), and potassium ferrocyanide were purchased from Fisher Scientific (Dehradun, India). Gold salt (tetra chloroauric acid) was purchased from SRL (Mumbai, India), and potassium ferricyanide and sodium chloride were purchased from Central Drug House (New Delhi, India). EDC (ethyl (dimethylamine propyl) carbodiimide), NHS (N-hydroxy succinimide), and DI water were obtained from Sigma Aldrich (India). The amine-modified DNA sequences were purchased from Gene Bo Solutions (Dehradun, India). As previously reported, the DNA sequences were as below [5]:

- PCA3 Probe Sequences: TTTTTTTCCCAGGGATCTCTGTGCTTCC
- PCA3 Target Sequences: GGAAGCACAGAGATCCCTGGG

### 2.1. Synthesis of Au–GQD (Gold–Graphene Quantum Dot) Nanohybrid

Synthesis of the Au–GQD nanohybrid was performed via a reduction reaction of HAuCl_4_. Dextrose was used as a carbon source for the Au–GQD nanohybrid, tri-sodium citrate was used as a stabilizing agent, and gold (III) chloride trihydrate was also used. A standard home microwave was used to make the process easier. To initiate GQD synthesis, a 5 mL solution of 0.6 M D-glucose in deionized water was exposed to microwave irradiation at a consistent power of 800 W for 3–5 min. The resultant solution was then carefully extracted with 5 mL of 0.1 M tri-sodium citrate. Evidenced by the emergence of a pale-yellow color, it confirmed successful GQD synthesis. Afterward, at room temperature, 150 µL of aqueous chloroauric acid (HAuCl_4_ 3H_2_O) was introduced into the pre-formed GQD solution, leading to the synthesis of Au–GQD nanohybrid. This approach synthesized Au–GQD nanohybrid using pre-formed GQDs that reduced HAuCl_4_ 3H_2_O to gold nanoparticles upon the outermost layer at ambient temperature with ionic interaction. The final product obtained was a 5 mL solution of an Au–GQD nanohybrid, encapsulating the synergistic properties of gold and graphene quantum dots [23].

### 2.2. Characterization of Au–GQD Nanohybrid

A UV spectrophotometer (Shimadzu’s UV-VIS 1900, Japan) was used to characterize the Au–GQD nanohybrid. The UV spectra presented characteristic absorption peaks of Au–GQDs at around 524 nm and 280 nm. Fourier transform infrared spectroscopy (FTIR; PerkinElmer Frontier) showed the presence of –COOH on the surfaces of the GQDs. Scanning electron microscopy (SEM, FEI Quanta 450, Oregon, USA) was performed on the sample of the synthesized Au–GQD nanohybrid. It presented uniform surface morphology and size distribution all over the surface. The X-ray powder diffraction (XRD, Malvern Panalytical Empyrean Series 3) pattern of the synthesized nanohybrid indicated the presence of gold and GQDs.

### 2.3. Electrodeposition of Au–GQDs on ITO

The electrochemical deposition offered outstanding purity, enhanced adhesion, and homogenous layer growth on electrode surfaces. An increase in the electrochemically active surface area could have improved the kinetics of the electron transport. These advantages led to the selection of the electrodeposition methods for creating hybrid graphene-based nanoplatforms, and most electrodeposition methods have been used for bioanalytical sensors [24,25]. Electrodeposition was performed using a 3-point electrode system of ITO, platinum wire, and Ag/AgCl (working electrode (WE), counter electrode (CE), and reference electrode (RE), respectively). The ITO was washed for 15 min by sonication with ethanol and DI water. The cleaned ITO electrodes were appropriately dried, and an Au–GQD hybrid nanomaterial solution was prepared in the ferri–ferrocyanide electrolyte solution at a ratio of 24:1 and electrodeposited, layer by layer, through CV within the potential range of −1.4 to 0.6 V, with a scan rate of 50 mV/s for a range of cycles (3–9). The resulting CV scans formed a thin film of hybrid nanomaterial on the ITO surface, ranging from light blue to dark blue depending on the number of cycles performed [26,27].

### 2.4. Surface Immobilization of DNA Probes and Electrochemical Analysis

The electrodeposited ITO/Au–GQDs were initially functionalized using 20 µL of freshly prepared 0.5 M EDC and 0.2 M NHS to trigger the –COOH functional group on the GQDs. This functionalized surface provided a functional group to the PCA3 DNA probe for forming NHS ester bonds. This created a monolayer of a PCA3 ssDNA probe with an –NH_2_ group at the beginning of the sequence. The NH_2_ group of the PCA3 probe reacted with the NHS ester and formed a covalent bond with the GQDs [28]. This functionalization was confirmed using FTIR. Further, electrochemical measurements were performed using a three-electrode setup in Autolab (multiautolabM204, USA), in which ITO/Au–GQDs/probe was used as the working electrode, Ag/AgCl and platinum wire was used as the reference and counter electrodes. The electrolyte was 0.1 M phosphate buffer saline/ferro–ferricyanide (pH 7.0). The initial analysis and PCA3 monitoring of ITO and the Au–GQD/PDNA characterization were executed by employing CV at a range of −1 to 1 V at 50 mV/s and EIS at a range of 0.1 Hz to 0.1 MHz. Au–GQD electrodeposition was executed by performing CV in the potential range of −1.4 to +0.4 V for different cycles (3, 5, 7, 9). All experiments were performed multiple times (n = 3) using various electrodes. The scan rate study employed an extensive range from 20 to 100 mV/s. Fabricated sensor optimization used CV from −1 to 1 V at 50 mV/s. Probe DNA concentrations were taken from a wide range of probe DNA (PDNA) concentrations (10–50 µM), and the incubation time was optimized for successful binding with the probe and target DNA (TDNA) molecules (5–30 min). The electrolyte pH range was taken (6.0 to 8.0). A PCA3 target DNA probe with a comprehensive concentration range of 1 µM–100 fM and a volume of 10 µL was used. A shelf-life study of the sensor was also performed for up to 19 ± 1 days.

## 3. Results

### 3.1. Nanohybrid Characterization

#### 3.1.1. Ultraviolet–Visible Spectroscopy (UV–Vis)

**Figure 2:**
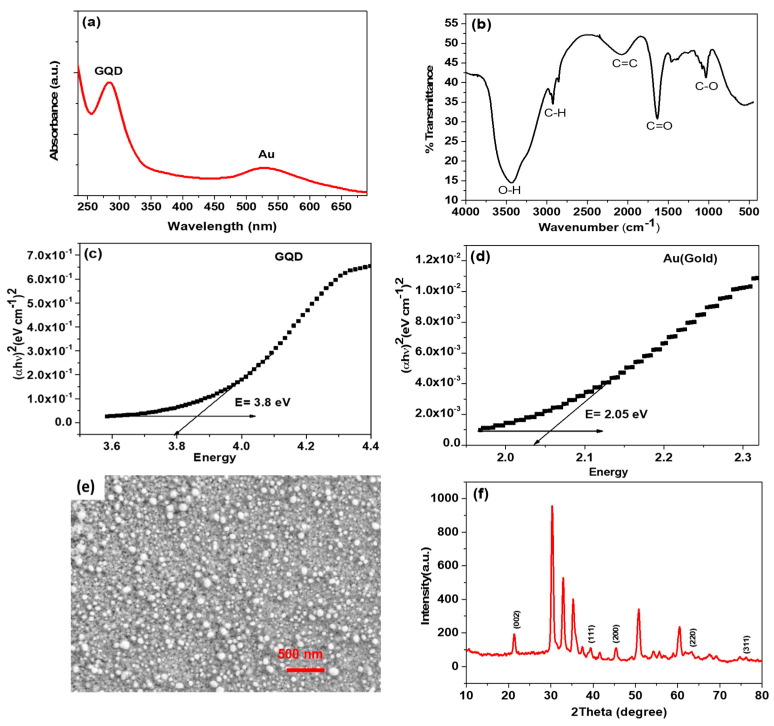
(**a**) UV–visible spectrophotometer of Au–GQDs. (**b**) FTIR spectra of synthesized nanohybrid Au–GQDs. (**c**) The band gap of the GQDs. (**d**) The band gap of Au. (**e**) SEM image (scale 500 nm) of the modified electrode with Au–GQD nanohybrid (ITO/Au–GQD). (**f**) XRD pattern of Au–GQDs, presenting the Au peak at 23.60° and peaks at 30.50°, 31.80°, 35.10°, 50.80°, and 60.0° due to ITO.

The peak was found at 286 nm in the UV–visible spectrum due to the light absorbed by the graphene quantum dots (GQDs). The detected peak at 532 nm represented the light captured by the gold (Figure 2a). These peaks demonstrate the practical synthesis of Au–GQDs [18]. In Figure 2c,d, the calculated band gaps of GQD and Au are 3.8 eV and 2.05 eV, respectively. A reported study states that a decreasing energy band gap is associated with larger GQDs, proving a correlation between energy band gap and material size [29,30].

#### 3.1.2. Fourier Transform Infrared (FTIR)

In the FTIR spectra of the Au–GQD nanocomposite, the O–H bond stretching was accountable for the large peak at 3443 cm^−1^, which was due to the absorbance of water by the nanocomposite. Considering that alkane was present, the C–H stretching at 2922 cm^−1^ was expected. The C=C bond (alkene) was expected at 3100 cm^−1^, but due to the electropositive (Au^+^) atom, it was shifted to 2069 cm^−1^ (Figure 2b). The expected carbonyl stretching frequency could be obtained at 1700–1725 cm^−1^, but due to the presence of cationic Au, this value was shifted from the expected value to 1634 cm^−1^. The C–O bond frequency was obtained with 1036 cm^−1^ as its characteristic peak [31].

#### 3.1.3. Scanning Electron Microscopy (SEM)

As-deposited Au−GQD/ITO is shown in the Figure 2e SEM images. The SEM image of the ITO/Au–GQDs reveals that the Au–GQD particles were electrodeposited on the ITO’s surface and had a roughly sphere-like form. Furthermore, more prominent clusters formed from the aggregation and accumulation of the Au–GQD particles. The measured average particle size was 25 ± 5 nm. This showed a uniform distribution of the smaller-sized nanohybrid on the surface of the electrodes, showing that they had electroactive and absorbent sites. This modification extends electrodes’ surface areas and charge transfer rates [32,33].

#### 3.1.4. X-ray Powder Diffraction (XRD)

The XRD spectrum of the synthesized Au–GQD nanohybrid displayed distinctive diffraction peaks and differences in peak intensities (presented in Figure 2f). Due to the (002) plane, the primary diffraction peak of the GQDs was seen at around 23.60°. Likewise, many diffraction peaks, at 38.0°, 64.30°, and 76.90°, corresponded to AuNPs, while the remaining peaks at 30.50°, 31.80°, 35.10°, 50.80°, and 60.0° were caused by the surface of the ITO substrate [34,35].

### 3.2. Electrochemical Characterization of Electrode Fabrication Stages

**Figure 3:**
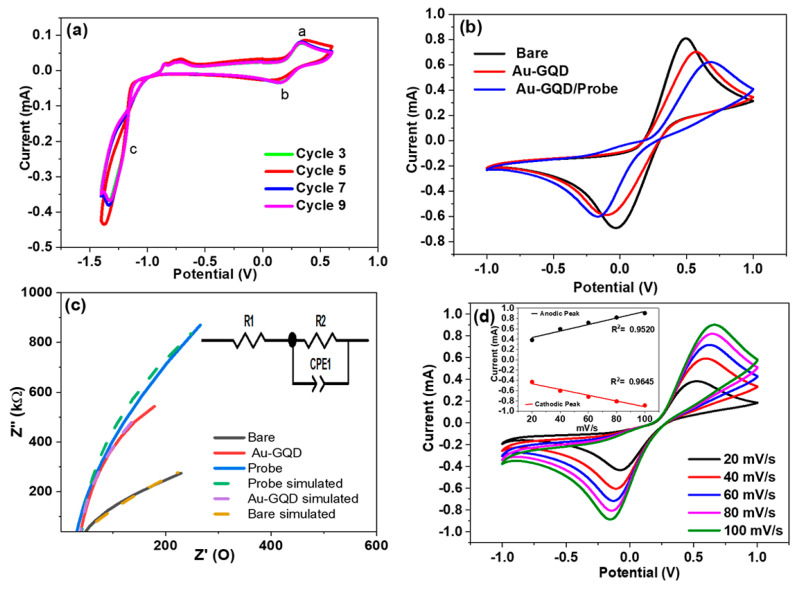
(**a**) Electrodeposition of Au–GQDs by cyclic voltammetry. (**b**) Cyclic voltammogram of bare ITO, ITO/Au–GQDs, and ITO/Au–GQDs/probe. (**c**) Nyquist plot of bare ITO, ITO/Au–GQDs, and ITO/Au–GQDs/probe (inset: Randel’s circuit simulation was fitted with a curve). (**d**) Voltammogram of scan rate study of ITO/Au–GQDs/probe (inset: linear plot of anodic peak and cathodic peak).

CV analysis of the electrodeposition of Au–GQDs on the ITO surface is presented in Figure 3a. Here, two cathodic peaks and one anodic peak can be observed, which shows the CV electro-analysis curves of the Au–GQDs on the ITO. The peak currents improved with each subsequent potential scan, indicating that the Au–GQD hybrid was effectively reduced electrochemically on the electrode surface. The current peak (c) suggests a reduction in the oxygen-containing group on the GQDs; the peak current observed in a and b was caused by the reaction of a specific redox on the graphene surface [27,36]. The obtained voltammograms are shown in Figure 3b. Bare ITO, ITO/Au–GQD, and ITO/Au–GQD/probe interfaces were previously defined by the CV, and EIS observations were carried out in the ranges of −1.0 to 1.0 V at 50 mV/s and 0.1 Hz to 0.1 MHz. The CV measurements of all the experiments were performed with 50 mv/s, within a potential range of −1 to +1 V, in the ferri–ferrocyanide electrolyte solution (Figure 3d). The Nyquist spectrum of Figure 3c aims to complement the voltammogram; the graph shows different processes happening at the electrode–electrolyte interfaces.

Consequently, the bare ITO exhibited less resistance, whereas with the ITO/Au–GQDs and ITO/Au–GQDs/probe (after modification with nanohybrid and the probe), the resistivity of the electrode interfaces increased, which complements the voltammogram in Figure 3b. The higher resistance from immobilizing the Au–GQD nanohybrid is linked to the electrostatic repulsion between the –COOH groups on the tiny particles, which serves as a barrier layer and prevents electrons from transferring from the redox probe to the electrode. Additionally, large linker molecules hampered the electron transfer route and induced steric hindrance.

## 4. Discussion

### 4.1. CV and EIS Responses

The data obtained from the EIS generated the Nyquist plot, and the plot curve fitting was performed using Z-view software. The curve fitting and circuit parameter estimation were performed using Randel’s circuit simulation. The simulated circuit and simulated graph are presented in the Figure 3c inset. R_1_ represents the electron transfer resistance among the working electrode and counter electrode, and R is the electron transfer resistance of the working electrode surface. The half-circular arcs appear depressed, a sign of a non-uniform double-layer capacitor (modeled employed constant phase element (CPE)). This demonstrates the various interfacial processes occurring at the electrode–electrolyte contact. The estimated values for the Randel’s circuit components used to model the simulated circuit are presented in Table 1. From Table 1, it can be seen that the values of R_1_ and R_2_ increase along with a slight change in the constant phase element (CPE) (it shows the electrochemical double-layer capacitance of the electrode) at the electrode–electrolyte interfacial after immobilization of the Au–GQDs and the PCA3 probe as compared to bare ITO. As displayed in Figure 3b, it is evident that the ITO/Au–GQDs and ITO/Au–GQDs/probe show the lowest currents. It might have happened that the electrode’s electrochemical behavior changed when the Au–GQD nanohybrid was applied to the ITO electrode rather than the bare ITO. In the instance of the Au–GQD nanocomposite, the lack of current or decreased current refers to a decreased charge transfer mechanism, most likely carried on by the electronic or steric effects of the nanocomposite. The current response of the ITO electrode was 0.8 mA due to the high conductivity of the ITO layer, which showed a decrease (0.7 mA) after coating the Au–GQD hybrid on its surface, attributed to the thin film of nanocomposite decreasing the current response due to the comparatively less conductive behavior of the GQDs. The Au nanoparticles exhibited higher electrical conductivity, but after the combination with GQD, there was a hindrance in the movement of the electrons from the negatively charged electrolyte solution to the electrode surface due to the presence of negatively charged –OH and –COOH groups in the GQDs. Further, a decreased current response was observed after surface functionalization, with a DNA probe of up to 0.6 mA due to the charged backbone of a single-stranded DNA sequence made up of sugar and phosphates having many negative charges exposed on the surface [37]. As presented in Figure 3d, these CV curves demonstrate rising peak current values as the scan rate increases (20–100 mV/s). Furthermore, the ideal diffusion-controlled process of the reversible system can be observed in the distance between the peak potential values and the CV curve shapes. Additionally, this study confirmed electrode stability; an additional plot of peak current against the square root of the scan rate was drawn to confirm the reversibility and diffusion-controlled characteristics of the redox probe, presented in the Figure 3d inset. Further, the calibration curve of the scan rate study had R^2^ values of 0.9520 and 0.9645 for the anodic and cathodic peak values, respectively [38].

### 4.2. Optimization Studies

**Figure 4:**
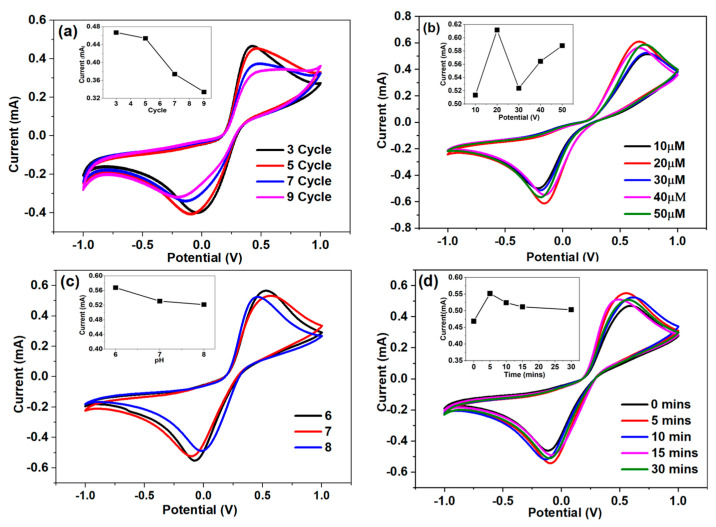
(**a**) Voltammogram of electrodeposition cycle optimization. (**b**) Cyclic voltammetry of probe concentration optimization. (**c**) pH optimization. (**d**) Incubation time.

The developed sensor was optimized over various parameters, including electrodeposition cycle, PDNA concentration, pH, and incubation time, as displayed in Figure 4. The current response decreased when the layer of Au–GQDs on the ITO surface increased, according to the corresponding Figure 4a. In this study, three layers (cycles) of Au–GQDs on ITO showed the highest current responses, which is why these three cycles were chosen for further research. The I_pa_ was dramatically reduced when the PDNA concentration was increased from 10 to 50 µM, as shown in Figure 4b. This was caused by the negatively charged phosphate backbone of the PDNA and the negatively charged electrolyte. This combination inhibited the electron transfer flow. Whenever the PDNA concentration decreased to 20 µM, it led to higher electron movement (increased I_pa_).

Consequently, to conduct further studies, the PDNA concentration of 20 µM was found to be the optimum concentration. The kinetics on the sensor surface were affected by pH (6.0 to 8.0), as shown in Figure 4c. In this investigation, there was little change in the current of the oxidation peaks from pH 6.0 to 8.0. As the biological samples worked well at pH 7.0, we picked the 7.0 pH value as optimal for further studies. The hybridization process began after the PDNA-modified electrodes were coated correctly with 10 µL of 1 µM TDNA, and the hybridization period was adjusted from 1 min to 10 min under optimized conditions, as shown in Figure 4d. When the hybridization duration was increased from 5 min to 30 min, the current response decreased from 0.55 mA to 0.50 mA, indicating that the two single-stranded DNA molecules were successfully hybridized to form a double-helix structure [39]. However, after 10 min, the I_pa_ exceeded saturation, demonstrating that the PDNA and TDNA strands had hybridized entirely.

### 4.3. PCA3 Detection Using ITO/Au–GQD/PDNA Electrodes

**Figure 5:**
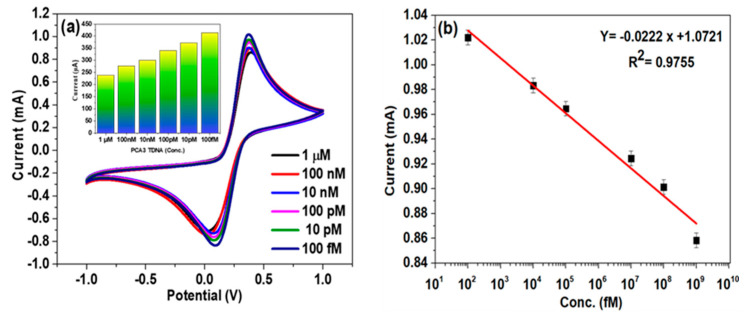
(**a**) Voltammogram at different TDNA concentrations; inset: the graph showing the relation between concentration and current response by changing the y-axis unit. (**b**) The straight line of TDNA concentration.

Using CV, the analytical properties of the ITO/Au–GQD/PDNA electrodes were investigated in the potential range of −1.0 to 1.0 V (50 mV/s). There were different PCA3 TDNA concentrations taken from 1 µM to 100 fM. Figure 5a highlights the corresponding graphs.

The voltammograms disclose that adding PCA3 TDNA repeatedly caused a drop in the peak anodic currents (I_pa_), as displayed by a nearly twofold decrease at 1 µM TDNA from the response at 100 fM TDNA in Figure 5a. Additionally, we used the change in the y-axis (µA) and subtracted the background (ITO/Au–GQD/probe) from the current values (ITO/Au–GQD/probe/target) to plot the graph presented in Figure 5a, inset. At a concentration of 100 fM, the I_pa_ was measured to be 1.02 mA, while at 1 µM, it decreased to 0.84 mA due to the pairing of the probe and target DNA, which produced a substantial double helix structure [40]. The load of negatively charged moieties on the biosensor surface increased, significantly inhibiting the electron flow, and hence, the current response showed a decreased current response for the TDNA. The measurement calibration plot for the PCA3 concentration versus the I_pa_ response was plotted, as shown in Figure 5b. The graph shows linearity among the I_pa_ and TDNA concentrations from 1 µM to 100 fM of TDNA concentration. Furthermore, using the equation LoD = 3 SD/m (where SD stands for standard deviation and m for the slope of the calibration plot), it was determined that the detection limit was 211 fM. The proposed biosensor’s LoD was calculated by applying the slope value from Figure 5b, which displays a regression equation with an R^2^ value of 0.9755. The presented results indicate that the developed sensor exhibited an overwhelming LoD, which presented an improvement over the other developed electrochemical sensors published earlier (Table 2). The developed Au–GQD/PDNA-based sensor exhibited femto-molar-level LoD throughout a broad PCA3 TDNA concentration range (1 µM to 100 fM). This improvement was due to the effective immobilization of the PDNA onto the Au–GQD-modified electrodes through strong carbodiimide coupling and ballistic electron transit across the interface between the electrodes and the electrolyte, which are crucial for calibrating any sensing device.

### 4.4. Selectivity and Shelf-Life Studies

**Figure 6:**
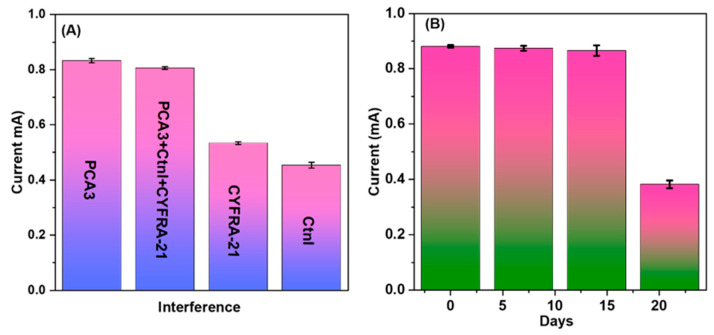
(**A**) Demonstration of the interference study with different TDNAs. (**B**) The shelf life of the developed sensor was up to 19 ± 1.

The fabricated sensor effectively recognized PCA3 TDNA when mixed with other target DNAs, such as CYFRA–21 and CtnI (Cardiac Troponin) DNA. A total of 1 µM non-complementary DNA (CYFRA–21 and CtnI) and PCA3 TDNA was employed for the selectivity assessment, and compatible I_pa_ values were observed at the calibrated potential. The current response produced by the PCA3 TDNA (0.77 mA) showed a slight change when combined with CYFRA–21 and CtnI. Furthermore, the CYFRA–21 and CtnI TDNA alone exhibited I_pa_ values of 0.53 mA and 0.45 mA, respectively, as seen in Figure 6A. The shelf life of the pre-immobilized sensor was also quantified (Figure 6B), and it presented an agreement of sensing with the highest sensitivity performance for up to 19 ± 1 days. This showed the value of I_pa_ 0.9 mA at 1 µM TDNA over 21 days at 0.38 mA. Later, it presented a considerable reduction in I_pa_ value, demonstrating the sensor’s reliability and consistency over 19 ± 1 days.

## 5. Conclusions

The presented work demonstrates the development of a portable electrochemical DNA biosensor for the rapid diagnosis of prostate cancer. A unique Au–GQD nanohybrid was used to facilitate the capture of PCA3 DNA biomarkers with an LoD of up to 211 fM. The sensor demonstrated a good linear nature within TDNA concentrations of 1 µM to 100 fM. The developed biosensor has a shelf life of up to 19 ± 1 days and a detection time of 5 min. The fabricated sensor presented improved sensing, response time, and stability in comparison with other developed sensors in the literature. The developed sensor and methodology are potentially eligible for commercialization and to be used in rapid testing and screening for prostate cancer patients. The presented work demonstrates the development of a portable electrochemical DNA biosensor for rapidly diagnosing prostate cancer. A unique Au–GQD nanohybrid facilitates the capture of PCA3 biomarkers with a LoD of up to 211 fM. The sensor demonstrated an excellent linear nature within TDNA concentrations of 1 µM to 100 fM. The developed biosensor has a shelf life of up to 19 ± 1 days and a detection time of 5 minutes. The fabricated sensor presents improved sensing, response time, and stability compared to other developed sensors in the literature. The developed sensor and methodology are potentially eligible for commercialization and to be used in rapid testing and screening for prostate cancer patients.

## Figures and Tables

**Table 1:** Assessment of various interfacial characteristics of the simulated circuit using Z-view software.

Stages	R_1_(Ω)	R_2_(Ω)	CPE1-T(F)	CPE1-P(F)
Bare	24	2200	0.061	0.71
Au–GQD modified	37	2900	0.064	0.98
Probe modified	40	3800	0.065	0.99

**Table 2:** Comparison of the proposed biosensors for prostate cancer-sensing properties with some previously published research.

S.N.	Surface Modifier	Linear Range	Sensitivity (LoD)	Ref.
1	ZnO tetrapods	1 pM to 50 µM	1 pM	[41]
2	Screen printed gold electrode	0.01nM to 100 nM	0.4 pM	[42]
3	SPECs	25 pM to 10 nM	4.4 pM	[43]
4	Al-IDE	10 pM to 100 nM	10 pM	[44]
5	Sox-nps	0.1 µM to 100 µM	0.10 pM	[45]
**6**	Gold–graphene quantum dots	1 µM to 100 fM	211 fM	**This work**

## Data Availability

Data will be available on request to the corresponding author.
